# An Optimized Chloroplast DNA Extraction Protocol for Grasses (Poaceae) Proves Suitable for Whole Plastid Genome Sequencing and SNP Detection

**DOI:** 10.1371/journal.pone.0002813

**Published:** 2008-07-30

**Authors:** Kerstin Diekmann, Trevor R. Hodkinson, Evelyn Fricke, Susanne Barth

**Affiliations:** 1 Teagasc Crops Research Centre, Carlow, Ireland; 2 Department of Botany, School of Natural Sciences, Trinity College Dublin, University of Dublin, Dublin, Ireland; Purdue University, United States of America

## Abstract

**Background:**

Obtaining chloroplast genome sequences is important to increase the knowledge about the fundamental biology of plastids, to understand evolutionary and ecological processes in the evolution of plants, to develop biotechnological applications (e.g. plastid engineering) and to improve the efficiency of breeding schemes. Extraction of pure chloroplast DNA is required for efficient sequencing of chloroplast genomes. Unfortunately, most protocols for extracting chloroplast DNA were developed for eudicots and do not produce sufficiently pure yields for a shotgun sequencing approach of whole plastid genomes from the monocot grasses.

**Methodology/Principal Findings:**

We have developed a simple and inexpensive method to obtain chloroplast DNA from grass species by modifying and extending protocols optimized for the use in eudicots. Many protocols for extracting chloroplast DNA require an ultracentrifugation step to efficiently separate chloroplast DNA from nuclear DNA. The developed method uses two more centrifugation steps than previously reported protocols and does not require an ultracentrifuge.

**Conclusions/Significance:**

The described method delivered chloroplast DNA of very high quality from two grass species belonging to highly different taxonomic subfamilies within the grass family (*Lolium perenne*, Pooideae; *Miscanthus*×*giganteus*, Panicoideae). The DNA from *Lolium perenne* was used for whole chloroplast genome sequencing and detection of SNPs. The sequence is publicly available on EMBL/GenBank.

## Introduction

Chloroplasts are a form of plastid, derived originally from independent living cyanobacteria that were incorporated into plant cells during evolution [1], [2], [3]. Up to 300 chloroplasts [4] can be found in one plant cell where they are the location of photosynthesis. In most species chloroplasts are usually strictly maternally inherited [5]. Due to their endosymbiotic origins chloroplasts contain their own genome that generally shows highly conserved features regarding gene content and gene order between species. Thus, chloroplast genome sequences are important to improve knowledge about the fundamental biology of plastids, to help understand evolutionary and ecological processes in plants, to facilitate the development of biotechnological applications (e.g. plastid engineering), and to improve the efficiency of breeding schemes.

To date, more than 100 complete chloroplast genome sequences of land plant species are publicly available (http://www.ncbi.nlm.nih.gov/genomes/ORGANELLES /plastids_tax.html) and most are from angiosperms, especially eudicots (60 sequences) and monocots (17 sequences). Many agricultural plant species belong to either of these two groups but the monocot grass family Poaceae is by far the most important globally from a socio-economic perspective as it contains the cereals, forage species and several other economic crops [6]. Plastid genome sequences from this family are of high importance and are needed for a whole range of applications.

Plastid genome sequences offer new engineering targets for biotechnology as transgenes are normally integrated into intergenic spacer regions. Therefore it is necessary to know the exact chloroplast genome sequence of the target species to design species-specific vectors to ensure the engineering success [7]. So far, for example, genes for insect [8], herbicide [9] or disease resistance [10] have been integrated into the tobacco chloroplast genome and might also be useful for integration into the chloroplast genome of Poaceae species. To date, plastid engineering has been successfully optimized for only one monocot species, *Oryza sativa*, of the grass family (Poaceae) [11], [12]. Furthermore detailed characterization of plastid haplotypes is essential for thorough studies of the population genetic, phylogenetic and taxonomic background of grass species [13]. Chloroplast DNA (cpDNA) sequences are also important for conventional plant breeding programmes to characterize and hence manipulate organelle genomes by breeding. Besides, they are of interest to investigate nucleo-cytoplasmic effects [14] since plastid signals controlling nuclear gene expression can have both positive and negative effects on gene expression [15].

Complete cpDNA sequences are in general obtained by sequencing either cpDNA clones found as ‘contaminations’ in genomic libraries [e.g. 16] or by sequencing high purity extracted cpDNA that has been cloned into sequencing vectors [e.g. 17]. Recently a primer walking strategy, based on consensus cpDNA sequencing primers (CCSPs) designed from the chloroplast genome of *Nicotiana tabacum* in comparison with the chloroplast genome of *Arabidopsis thaliana* and *Spinacia oleracea,* was used for obtaining complete chloroplast genome sequences [18]. We were especially interested in the chloroplast genome of perennial ryegrass (*Lolium perenne* L.) which is also a member of Poaceae and one of the most important forage crops in Europe.

We aimed to sequence the complete cpDNA genome of *L. perenne* and therefore chose to extract pure cpDNA from a perennial ryegrass population and to sequence it using a shotgun sequencing approach. We used this approach because it is cost and time effective and it allowed us to assess nucleotide variation within the chloroplast genome of a cross pollinating grass species by documenting the occurrence of single nucleotide polymorphisms (SNPs). SNPs are useful to detect chloroplast haplotype variation in individual plants of a cultivar and to help identify cytoplasmic gene pools. They can be used to evaluate the mutation rate in chloroplast genomes and to detect sequences belonging to mitochondrial or nuclear DNA with a high similarity to the chloroplast genome (e.g. greater than 98 %) [19]. Therefore SNPs might also help to obtain information about horizontal gene transfer within a species.

Although general protocols for DNA extraction from plants are well developed [see 20 for a review], the success of cpDNA extractions is highly species dependent [21]. Most of the available protocols for cpDNA extraction were designed and established using eudicot plants such as the protocol developed by 22 for pea (*Pisum*). We tried several of these protocols [e.g. 21], [22], [23] but found that they were unreliable for application in grasses. Possibly because of the high fibre content of grass leaves, cpDNA yields obtained with these methods were not high or pure enough (Figure S1) for whole chloroplast genome sequencing using the shotgun sequencing approach. Therefore we combined and modified existing protocols to develop a simple, robust and inexpensive method for isolating high purity cpDNA from grasses. The method described in this paper is based on a combination, modification and extension of protocols from 21, 23 and 24. To test our method, we used material from a perennial ryegrass population and clonal material of one genotype from another agriculturally and economically important grass species *Miscanthus*×*giganteus* Greef et Deuter ex Hodkinson and Renvoize. They are each representatives of the two largest grass subfamilies [6]. *Lolium perenne* belongs to the Pooideae subfamily that also includes wheat (*Triticum*), barley (*Hordeum*), and rye (*Secale*) and uses the C_3_ photosynthetic pathway. *Miscanthus*×*giganteus* belongs to the Panicoideae subfamily that includes maize (*Zea*), *Sorghum*, sugarcane (*Saccharum*) and millets (*Pennisetum* and *Setaria*) and has C_4_ photosynthesis. *Miscanthus*×*giganteus* is under considerable attention as a potential biomass crop for sustainable energy production. *Miscanthus* is also an important forage species in Asia and is globally important for the horticultural trade.

We demonstrate that our method is efficient for extracting high purity and quantity cpDNA from both young (perennial ryegrass) and mature fully expanded highly fibrous (*Miscanthus*) leaves. We anticipate that the cpDNA isolation method reported here has the potential to work well with all grass species and that it would offer an improvement to previously available protocols for monocots. Most existing protocols [e.g. 21] involved the use of an ultracentrifuge. The presented modified method has furthermore the added benefit of requiring only common laboratory equipment.

## Results and Discussion

**Figure 1:**
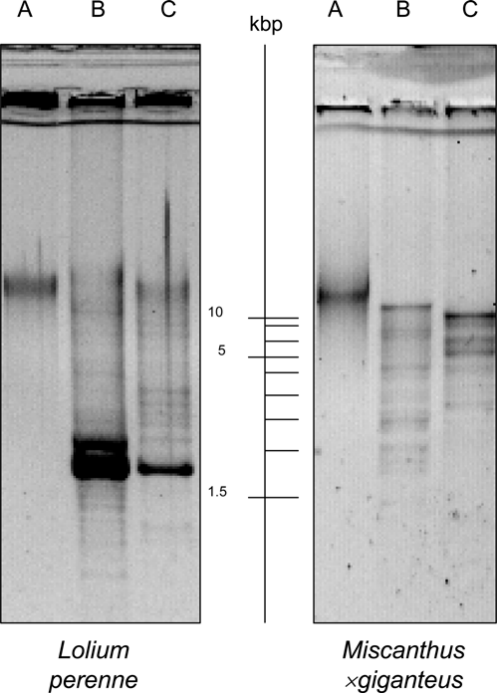
Digested cpDNA of *Lolium perenne* and *Miscanthus*×*giganteus* on a 1%-agarose-gel. (A = undigested, B = *Eco*RI digested, C = *Hind*III digested; mi-1 kb DNA Marker Go (Metabion international AG, Planegg-Martinsried, Germany).

Improved isolation methods for cpDNA from grass species are of high socio-economic value for a range of applications in this highly valuable family (Poaceae). In this study we have optimized a procedure to extract very pure *Miscanthus* cpDNA of high quantity (70 µg/100 g starting leaf material) and very pure *Lolium* cpDNA of lower quantity (0.3 µg/100 g starting material). For both species the successful isolation of the chloroplast genome was determined by restriction digestion of the DNA visualized by agarose gel electrophoresis. The fine banding patterns were, in both cases, similar to the fine cpDNA banding patterns from other species typical for digested cpDNA (Fig. 1). The latter *Lolium* extract was subject to whole genome amplification using the GenomePhi DNA amplification kit (Amersham Biosciences, Prod.-no.: 25-6600-01) following the manufacturer's instructions. The sequencing of the entire cpDNA of *Lolium* using a classical shotgun sequencing approach and its pre-assembly was carried out by a company (GATC Biotech AG, Konstanz, Germany). The result confirmed, once more, our presumption that the isolated DNA was *Lolium* cpDNA and it enabled us to assemble the entire chloroplast genome of *L. perenne*. The sequence in complete length produced with this method is available on EMBL, GenBank or DDBJ (Accession number: AM777385).

**Figure 2:**
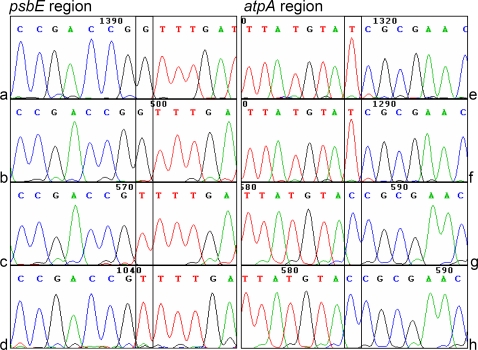
SNPs in the *psbE* and *atpA* region in the perennial ryegrass chloroplast genome (a, b, c, d = four different trace files of the same region in *psbE* / e, f, g, h = four different trace files of the same region in *atpA*).

Although protocols for sequencing cpDNA based on consensus primer walking of total genomic DNA exist [18], it is still essential to have protocols based on the more conventional approach of sequencing chloroplast DNA via highly purified cpDNA because it allows efficient detection of SNPs and insertion-deletion polymorphisms (Indels) within a plant population. Although chloroplast genomes are known to be in general highly conserved, a study of chloroplast microsatellite variation in *Lolium* [13] revealed high levels of chloroplast DNA haplotype variation in cultivated and wild accessions. Using the classical shotgun sequencing approach for sequencing the chloroplast genome of *Lolium* enabled us to detect 40 SNPs and 32 Indels in the cpDNA of the perennial ryegrass cultivar ‘Cashel’. SNPs were detected visually in the alignment files of the pre-assembled chloroplast genome of perennial ryegrass using the programme Lasergene (DNASTAR, Inc., Madison, USA). Two of them were, for example, in the *psbE* (7× genome coverage) and *atpA* (11× genome coverage) gene region, respectively. The minor genotype occurred twice (G, *psbE*) and four times (T, *atpA*), respectively (Fig. 2). In the *Lolium* chloroplast genome 80% of the SNPs and Indels found occurred in less than 40% of all tracefiles covering the respective region and could have remained unnoticed using a consensus primer walking approach. A similar situation was seen in rice cpDNA genome sequencing where a comparison of two different assemblies of the chloroplast genome of cultivar ‘Nipponbare’, the first available chloroplast genome of a monocotyledonous species, revealed a variation among the two sequences at 189 sites (79 SNPs/110 Indels)[19].

This cpDNA protocol has been developed and optimized for grasses by modifying existing protocols [21], [23], [24]. The main points at which the protocols differ are 1) the procedure to separate cell debris from intact chloroplasts, and 2) the procedure to purify the cpDNA by separating the chloroplasts from intact cells. Both steps were highly important because they reduced the amount of nuclear DNA contamination. Otherwise, intact cells and cell debris would be dissolved in the lysis step and nuclear and mitochondrial DNA would have been extracted in addition to the target cpDNA. Due to these two steps the protocol has the added benefit of not requiring the use of an ultracentrifuge as these are not available to many laboratories.

## Materials and Methods

### Plant material

*Lolium perenne* cv ‘Cashel’ was grown in soil under natural daylight and temperature conditions in a greenhouse. Young leaves, not older than 6 weeks and not taller than 10 cm, were harvested. Fully expanded *Miscanthus* leaf material was harvested from mature plants in a field plot and the tough midribs were manually removed prior to further processing.

### Reagents

**Buffer 1** after 24: 1.25 M NaCl, 50 mM Tris-HCl pH 8.0, 7 mM EDTA, 5% PVP-40, 1% BSA, 1 mM 2-mercaptoethanol; modifications: 0.1% BSA, 1 mM DTT instead of 2-mercaptoethanol.

**Buffer 2** after 24: 10 mM 2-ME, 10 mM Tris-HCl pH 8.0; 5 mM EDTA; 100 µg/ml Proteinase K; modifications: no 2-ME was used.

**Wash buffer** after 21: 0.35 M Sorbitol, 50 mM Tris-HCl pH 8.0, 25 mM EDTA.

**52% sucrose buffer** after 21: 52% sucrose in 50 mM Tris-HCl pH 8.0, 25 mM EDTA.

**30% sucrose buffer** after 21: 30% sucrose in 50 mM Tris-HCl pH 8.0, 25 mM EDTA.

**Lysis buffer** after 23: 20% sodiumsarcosinate, 50 mM Tris-HCl pH 8.0, 25 mM EDTA.

### Chloroplast isolation

**Figure 3:**
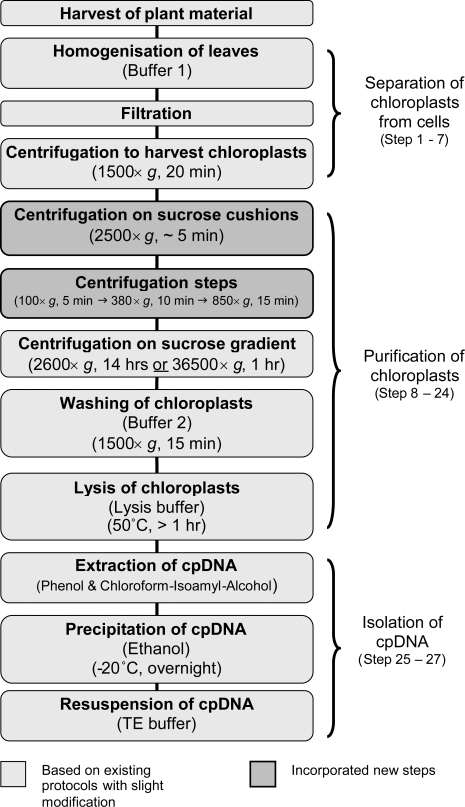
Flowchart showing the major steps for the isolation of high purity chloroplast (cp) DNA from grasses using the new optimised protocol.

All the following steps (summarized in Fig. 3) were carried out at 4°C if not otherwise stated. Leaves were cut in pieces (<1 cm) and homogenized in ice-cold **buffer 1** (up to 800 ml/100 g leaf material using an Ultra-Turrax T 25 basic (IKA) homogenizer). The homogenate was filtered twice through four layers of Miracloth (Merck), one time with squeezing and one time without squeezing, and afterwards centrifuged (1,366×*g*, 20 min). Each pellet was re-suspended twice in 100 ml of buffer 1 using a soft paintbrush and centrifuged (1,366×*g*, 20 min).

The resulting pellets were each dissolved in 25 ml **wash buffer**. 6 ml of the solution was carefully layered on a sucrose cushion consisting of 5 ml of **30% sucrose solution** in a 15 ml centrifuge tube and centrifuged (2,500×*g*, 5 min, swinging bucket rotor). This was the first step that differed from the other protocols. It was necessary for separating intact chloroplasts from cell debris, broken chloroplasts and other cell organelles. However, the resulting pellets still contained intact cells and a further step for removing them was required.

Therefore, each pellet was washed in 5 ml wash buffer and the centrifugation step was (2,500×*g*, 5 min) repeated. Two pellets were always combined, re-suspended in 5 ml wash buffer and centrifuged (2,500×*g*, 3 min). The resulting pellets were then re-suspended in 1.5 ml wash buffer, transferred to 2 ml microcentrifuge tubes and centrifuged (100×*g*, 5 min). The supernatant was transferred to new tubes and centrifuged at 380×*g* (10 min). This gradual centrifugation was the second additional inserted step into the isolation protocol to increase the cpDNA purity by separating the chloroplasts from intact cells. If larger amounts of starting material are used then a third centrifugation step at 850 g is necessary. The pellets from the last two steps were the purest with the smallest amount of cell debris.

The pellets were each dissolved in 1 ml wash buffer and 2 ml of the chloroplast solution was layered carefully on top of sucrose gradients, prepared by using 30% and **52% sucrose solution**, respectively. The two previous incorporated steps enabled the gradients to be run in a common laboratory centrifuge instead of an ultracentrifuge (2,600×*g*, 14.5 hours, swinging bucket rotor/or 36,500×*g*, 1 hour, Sorvall SH 80 rotor).

The chloroplasts were collected into 50 ml centrifuge tubes from the interphase of the 52% to 30% layers using a wide-bore pipette and diluted with wash buffer up to a volume of 45 ml. The tubes were centrifuged to pellet the chloroplasts (1,500×*g*, 15 min).

### Lysis of chloroplasts

Each pellet was re-suspended twice in 10 ml of **buffer 2** and centrifuged (1,500×*g*, 15 min). Pellets were dissolved for chloroplast lysis in 2 ml buffer 2 and 200 µl of Proteinase K (10 mg/ml) each. After two minutes at room temperature 1/5 volume of **lysis buffer** was added and the tubes were incubated at 50°C for at least 1 hour with gentle mixing.

cpDNA was extracted in an equal volume of saturated phenol (pH 8.0), well mixed, centrifuged (2,600×*g*, 20 min) and the upper clear aqueous phase transferred to new tubes. An equal volume of chloroform:isoamyl-alcohol (24∶1) was added twice, tubes were well mixed, centrifuged (2,600×*g*, 20 min) and the supernatant transferred to a new tube (13 with modifications: chloroform:isoamyl (24∶1) instead of chloroform). Finally the cpDNA was precipitated with 99% ethanol and re-suspended in 1× TE. The cpDNA samples were treated with 2 µl RNAse/250 µl and the isolation success was determined by restriction digestion using *Eco*RI and *Hind*III. The results were visualized on a 0.5× TBE 1% agarose gel (Fig. 1).

## Supporting Information

Figure S1 Extraction results obtained by using a single protocol and a combination of two protocols (A = undigested, B = HindIII digested, C = EcoRI digested; 1 = untreated, 2 = DNase treated; M = mi-1 kb DNA Marker Go (Metabion international AG, Planegg-Martinsried, Germany).
(0.21 MB TIF)
