# Supplementary figures and images for: An Optimized Chloroplast DNA Extraction Protocol for Grasses (Poaceae) Proves Suitable for Whole Plastid Genome Sequencing and SNP Detection
Source: PLoS One. 2008 Jul 30;3(7):e2813. doi: 10.1371/journal.pone.0002813 (PMC2474675; doi:10.1371/journal.pone.0002813)

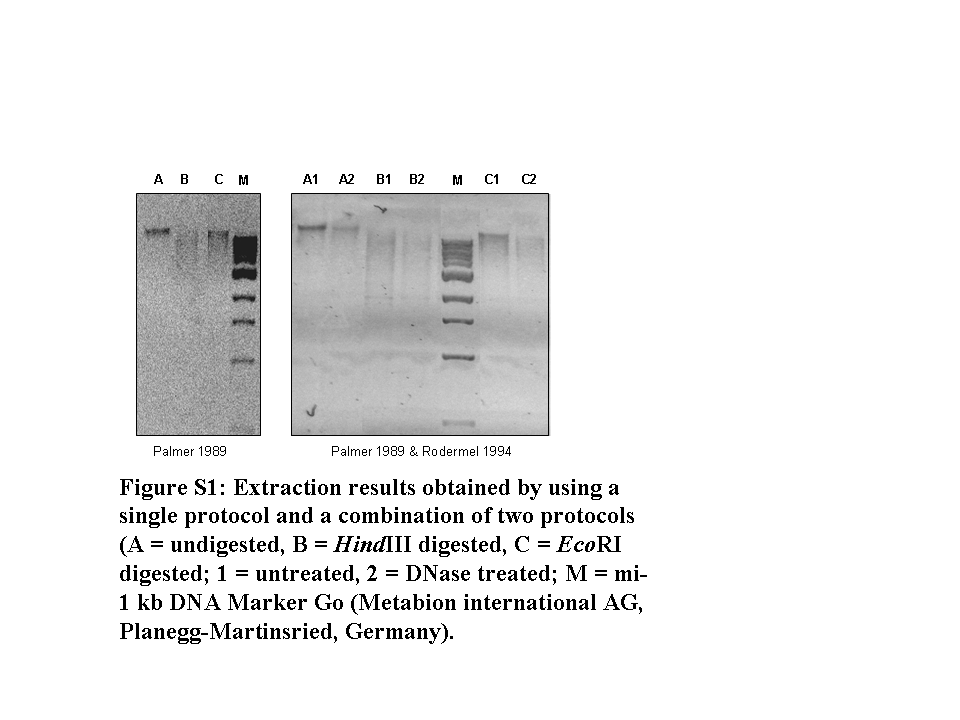

Supplement: Figure S1 — Extraction results obtained by using a single protocol and a combination of two protocols (A = undigested, B = HindIII digested, C = EcoRI digested; 1 = untreated, 2 = DNase treated; M = mi-1 kb DNA Marker Go (Metabion international AG, Planegg-Martinsried, Germany). (0.21 MB TIF) [file pone.0002813.s001.tif]
